# Antimicrobial Resistance and Infectious Complications in Cancer Immunotherapy: CAR-T Cells, Bispecific Antibodies and Immune Checkpoint Inhibitors

**DOI:** 10.3390/ijms27219907

**Published:** 2026-11-05

**Authors:** Angioletta Lasagna, Massimo Andreoni, Nicola Silvestris, Paolo Pedrazzoli

**Affiliations:** 1 Oncology Unit, Comprehensive Cancer Center, Fondazione IRCCS Policlinico San Matteo, 27100 Pavia, Italy; p.pedrazzoli@smatteo.pv.it; 2 Department of Systems Medicine, Infectious Disease Clinic, University of Rome Tor Vergata, 00133 Roma, Italy; andreoni@uniroma2.it; 3 Medical Oncology Department and Unit, IRCCS Istituto Tumori “Giovanni Paolo II”, 70124 Bari, Italy; silvestrisnicola@gmail.com; 4 Department of Internal Medicine and Medical Therapy, University of Pavia, Strada Nuova, 27100 Pavia, Italy

**Keywords:** CAR-T cells, bispecific antibodies, immune checkpoint inhibitors, infections, infection prevention, antimicrobial resistance, immunoglobulin replacement, vaccination

## Abstract

Cancer immunotherapies, including chimeric antigen receptor (CAR) T-cell therapies, bispecific antibodies (BsAbs), and immune checkpoint inhibitors (ICIs), have improved outcomes across hematologic and solid malignancies. Infection risk remains heterogeneous and depends on treatment platform, target antigen, lymphodepletion, immune reconstitution, concomitant immunosuppression, and supportive care. This narrative review synthesizes clinical trials, observational studies, and guidance identified through PubMed/MEDLINE, reference screening, and a targeted literature update through October 2026. It focuses on infection incidence, microbiological spectrum, antimicrobial resistance (AMR), multidrug-resistant (MDR) colonization and infection, antimicrobial exposure, prevention, and stewardship. Severe infections can be frequent with BCMA-directed BsAbs: the MagnetisMM-3 study reported grade 3–4 infections in 39.8% of elranatamab-treated patients, while a pooled global teclistamab cohort reported grade 3–4 infections in 55.3%; these estimates are study-specific and are influenced in part by COVID-19. Direct AMR evidence is concentrated in CAR-T and broader cellular-therapy cohorts, while comparable data for BsAbs and ICIs remain limited. Associations between broad-spectrum antibiotic exposure, microbiome disruption, and CAR-T outcomes are clinically important but remain vulnerable to confounding. Prevention should integrate immune status, prior microbiology, antimicrobial exposure, and local resistance patterns rather than infer resistance risk from treatment platform alone. Prospective studies using harmonized outcomes are needed to improve individualized infection prevention and antimicrobial stewardship.

## 1. Introduction

Over the past decade, cancer immunotherapy has profoundly transformed oncology and hematology. Immune checkpoint inhibitors (ICIs), which target PD-1, PD-L1, and CTLA-4, first showed that restoring endogenous antitumor immunity could induce durable clinical responses. More recently, chimeric antigen receptor (CAR) T-cell therapies and bispecific antibodies (BsAbs) have further expanded therapeutic options. Although these approaches are often collectively referred to as “immunotherapies,” they should not be considered immunologically equivalent. CAR-T cells require lymphodepleting conditioning and may induce prolonged B-cell aplasia, hypogammaglobulinemia, cytopenias, and profound perturbations of cellular immunity. BsAbs continuously engage endogenous T cells with tumor-associated targets and may produce sustained depletion of B cells or plasma cells, particularly with BCMA- and CD20-directed agents. ICIs, by contrast, primarily enhance pre-existing antitumor immune responses and usually do not cause profound immunodeficiency without additional immunosuppressive treatment [1,2,3].

Infectious risk should therefore be interpreted in relation to the specific immune defect induced by each platform. Relevant mechanisms include lymphodepletion, cytopenias, B-cell or plasma-cell depletion, hypogammaglobulinemia, toxicity-related immunosuppression, and prolonged exposure to healthcare environments. Patients receiving CAR-T cells or BsAbs may have extensive prior treatment, repeated hospitalization, central venous access, and recurrent antimicrobial exposure, which can increase the likelihood of colonization or infection with multidrug-resistant organisms (MDROs). In a retrospective CAR-T cohort, carbapenem-resistant organism (CRO) colonization was strongly associated with subsequent CRO infection, and CRO infection was associated with poorer one-year outcomes [4]. A separate mixed cellular-therapy cohort has also described concordance between rectal MDRO colonization and subsequent bloodstream infection, although most participants underwent allogeneic hematopoietic cell transplantation rather than CAR-T therapy [5]. These studies support incorporating previous microbiological results, recent antimicrobial exposure, healthcare exposure, and local epidemiology into empirical treatment decisions; they do not establish that resistance risk is equivalent across CAR-T, BsAbs, and ICIs. Broad-spectrum antibiotic exposure has also been associated with altered gut microbiome function and poorer outcomes after CD19-directed CAR-T therapy in observational cohorts [6,7], but confounding by disease severity and treatment-related factors remains possible. The present review therefore considers immune dysfunction, the temporal evolution of infectious risk, microbiological findings including AMR, and opportunities for individualized prevention and stewardship. The goal is to support patient-tailored clinical decisions without overstating the evidence available for any specific treatment platform.

## 2. Methods

A structured narrative literature search was conducted in PubMed/MEDLINE for the period January 2015 through May 2026, followed by a targeted update through October 2026 focused on AMR, antimicrobial prophylaxis, vaccine timing, and microbiome-related outcomes. Search terms combined controlled vocabulary and free-text terms for the therapeutic platforms (“CAR T”, “chimeric antigen receptor”, “bispecific antibody”, “T-cell engager”, “immune checkpoint inhibitor”, “PD-1”, “PD-L1”, and “CTLA-4”) with infection-related terms (“infection”, “bacterial”, “viral”, “fungal”, “Pneumocystis”, “CMV”, “HHV-6”, “antimicrobial resistance”, “multidrug-resistant”, “colonization”, “antibiotic exposure”, “microbiome”, “prophylaxis”, “vaccination”, “immunoglobulin”, and “hypogammaglobulinemia”). Studies were considered when they reported clinically relevant information on infection incidence, microbiology, risk factors, diagnosis, prevention, or management. Original clinical studies and current consensus guidance were prioritized for quantitative estimates and practical recommendations; relevant reference lists were also screened manually. Given the narrative design, studies were selected according to clinical relevance and their contribution to the domains addressed, rather than through a prespecified systematic-review protocol.

This approach has important limitations. The review is based principally on a single database and a narrative, relevance-based selection process, so selection bias and omission of eligible studies are possible. No formal risk-of-bias assessment, quantitative synthesis, or duplicate independent study selection was performed. The included studies also differ in malignancy, treatment exposure, infection definitions, prophylaxis, and follow-up. These limitations are particularly relevant to AMR, for which direct evidence in BsAb- and ICI-treated populations is sparse and much of the available evidence comes from CAR-T or broader hematology/cellular-therapy cohorts. The findings should consequently be interpreted as a clinically oriented synthesis rather than a systematic estimate of comparative risk.

## 3. Epidemiology of Infections Associated with Immunotherapies

Infection incidence varies considerably among immunotherapy platforms and individual studies. Reported rates are not directly comparable because underlying malignancies, prior treatment exposure, treatment duration, prophylactic strategies, follow-up, infection definitions, severity grading, and calendar periods differ. COVID-19 waves and changes in testing, vaccination, and supportive care have materially influenced some trial estimates, particularly in heavily pretreated myeloma cohorts. Longitudinal and comparative analyses have described the dynamic infection timeline after CAR-T and the differences reported between CAR-T and BsAbs [8,9]. The purpose of the following sections is therefore to describe the clinical context of available estimates, not to establish a definitive ranking of infection risk across therapies.

### 3.1. Bispecific Antibodies

Clinical studies of BsAbs in multiple myeloma (MM) report frequent infections, but the proportion of severe events varies substantially by agent, cohort, follow-up, and infection definitions [10,11,12,13]. In the phase 2 MagnetisMM-3 study, infections occurred in 69.9% of patients receiving elranatamab, with grade 3–4 infections in 39.8% and fatal infections in 6.5% [13]. In the global teclistamab cohort reported in a 2026 pooled analysis, infections occurred in 81.1% of patients and grade 3–4 infections in 55.3%; COVID-19 accounted for a substantial proportion of events [12]. Together, these studies show that grade 3–4 infections can exceed 40% in reported BCMA-directed BsAb cohorts; estimates remain cohort-specific and should not be generalized across agents or populations. CD20 × CD3 BsAbs, including mosunetuzumab, glofitamab, and epcoritamab, have different safety profiles in lymphoma cohorts and should be described separately rather than compared directly with heavily pretreated MM populations [14]. Infection risk in MM is shaped by baseline immune dysfunction, previous therapies, hypogammaglobulinemia, the treatment target, ongoing exposure, and prophylaxis. For BsAbs studied in solid tumors, available estimates are agent- and tumor-specific; the current evidence does not justify a single broad infection-rate range or a firm comparison with hematologic indications without specifying the trial, follow-up, and severity definitions [15].

### 3.2. CAR-T Cell Therapy

In adult patients with relapsed or refractory diffuse large B-cell lymphoma (DLBCL), or B-cell acute lymphoblastic leukemia (B-ALL), overall infection rates range from 30% to 50%, with grade ≥ 3 infections reported in 15–25% of patients [16]. In MM, BCMA-targeted CAR-T therapies, such as idecabtagene vicleucel (idecel) and ciltacabtagene autoleucel (ciltacel), have demonstrated overall infection rates of 40–80%, with severe infections occurring in approximately 20% of patients [17,18]. In solid tumors, CAR-T therapy remains investigational. Early-phase studies have reported infections, but available estimates are based on small, heterogeneous cohorts and vary according to CAR construct, lymphodepleting regimen, patient population, follow-up, and adverse-event ascertainment. Therefore, current evidence does not support a reliable overall or grade ≥3 infection-rate range, nor a direct comparison with hematologic indications [19].

### 3.3. Immune Checkpoint Inhibitors

Unlike CAR-T cell therapies and several bispecific antibodies (BsAbs), immune checkpoint inhibitors (ICIs) do not usually cause profound or sustained immunosuppression when administered alone. Consequently, the risk of infection associated with ICI monotherapy is generally lower than that observed with treatments that directly impair immune cell populations. Clinical trials and pooled analyses have reported any-grade infections in approximately 10–20% of patients receiving ICIs, although the incidence varies according to tumor type, treatment regimen, follow-up duration, and methods of infection reporting [20,21,22]. Severe and opportunistic infections, such as *Pneumocystis jirovecii pneumonia* (PJP) and invasive fungal infections, are uncommon in patients receiving ICI monotherapy without additional immunosuppressive treatment. When these infections occur, they are more frequently associated with immunosuppression resulting from the use of corticosteroids or other immunosuppressive agents to manage immune-related adverse events (irAEs), rather than with the direct effects of ICIs themselves [20,21,22]. The infection risk is different when ICIs are administered in combination with chemotherapy. In a meta-analysis of 36 randomized trials involving approximately 21,451 patients, ICI-based combinations with chemotherapy were associated with higher infection rates than chemotherapy alone. Any-grade infections occurred in approximately 15.8% versus 10.7% of patients, respectively, while grade 3–5 infections occurred in 4.4% versus 2.4%, respectively [22]. These findings indicate that the addition of an ICI to chemotherapy may be associated with an increased infection burden; however, the contribution of the ICI itself cannot be separated from that of chemotherapy and other treatment-related factors. Therefore, these estimates should not be extrapolated to ICI monotherapy or considered representative of all ICI-containing regimens. See Table 1 for the comparison of infectious risk among immunotherapy platforms.

**Table 1:** Cross-platform comparison of infectious risk.

Platform	Typical Infection Burden	Predominant Pathogens	Highest-Risk Period	Major Mechanisms	Key Preventive Priorities
CAR-T	CD19: ~30–50%; BCMA MM: ~40–80%; grade ≥ 3 varies (~15–25% in some cohorts) [16,17,18,23,24,25,26]	Gram-negative/positive bacteria; respiratory viruses; CMV/HSV/VZV; Aspergillus/Candida; PJP	0–30 d and later during prolonged cytopenia/B-cell or plasma-cell aplasia	Lymphodepletion; ICAHT; B-cell/plasma-cell depletion; hypogammaglobulinemia; CRS/ICANS treatment	HSV/VZV and PJP prophylaxis; consider antibacterial/mold prophylaxis for selected high-risk patients; IgG/IGRT; risk-adapted CMV monitoring; vaccination [27,28]
BsAbs	Elranatamab: 69.9% any-grade/39.8% grade 3–4; pooled teclistamab cohort: 81.1%/55.3%; COVID-19 contributed [12,13]	Respiratory bacteria/viruses; HSV/VZV; CMV; PJP; Candida/Aspergillus	Early cycles and throughout continuous treatment	Persistent T-cell engagement; B-cell/plasma-cell depletion; hypogammaglobulinemia; cytopenias	PJP/antiviral prophylaxis; monitor IgG and replace when indicated; vaccinate where feasible; use prior microbiology/local resistance data [27]
ICIs	Usually ~10–20% any-grade during monotherapy in selected cohorts; severe events less common; estimates rise with chemotherapy or immunosuppression [20,21,22]	Respiratory bacteria/viruses; urinary pathogens; TB/HBV/VZV/CMV in selected patients; PJP/fungi mainly with immunosuppression	During irAEs and especially prolonged/high-dose immunosuppression	Secondary immunosuppression rather than intrinsic profound immunodeficiency	Screening and vaccination; minimize avoidable immunosuppression; PJP/fungal prophylaxis only when indicated; HBV management [22,29,30,31,32,33]

Abbreviations: BCMA: B-cell maturation antigen; BsAbs: Bispecific antibodies; CAR-T: Chimeric antigen receptor T-cell therapy; CD19: Cluster of differentiation 19; CD20: Cluster of differentiation 20; CMV: Cytomegalovirus; CRS: Cytokine release syndrome; HBV—Hepatitis B virus; HSV: Herpes simplex virus; ICAHT: Immune effector cell-associated hematotoxicity; ICANS: Immune effector cell-associated neurotoxicity syndrome; ICIs: Immune checkpoint inhibitors; IgG: Immunoglobulin G; IGRT: Immunoglobulin replacement therapy; MM: Multiple myeloma; PJP: *Pneumocystis jirovecii* pneumonia; TB: Tuberculosis; VZV: Varicella-zoster virus.

## 4. Immunopathogenesis of Infection

The infectious risk associated with cancer immunotherapy results from the interaction of therapy-specific immune defects, toxicity-management strategies, and patient- and disease-related factors.

### 4.1. Therapy-Induced Immune Defects

#### 4.1.1. Lymphodepletion and Cytopenias

Lymphodepleting regimens administered before CAR-T infusion can induce transient aplasia of hematopoietic progenitors, resulting in reduced production of neutrophils and monocytes. Neutropenia compromises innate immune defenses by impairing chemotaxis, phagocytosis, and microbial killing through reactive oxygen species. The severity and duration of cytopenias are influenced by previous treatments, the intensity of lymphodepletion, and treatment-related inflammatory stress, thereby extending the period of susceptibility to bacterial, viral, and opportunistic infections [34].

#### 4.1.2. Immune Effector Cell-Associated Hematotoxicity

Prolonged cytopenias are an important contributor to infection risk following immune effector cell (IEC) therapy. Unlike the transient hematologic suppression commonly observed during the early post-treatment period, immune effector cell-associated hematotoxicity (ICAHT) refers to persistent or recurrent cytopenias extending beyond the expected period of initial hematologic recovery. These may affect one or more blood cell lineages, resulting in neutropenia, thrombocytopenia, anemia, or multilineage cytopenia, and may increase patients’ susceptibility to infectious complications [35].

Persistent neutropenia is particularly relevant to the risk of bacterial and invasive fungal infections, whereas prolonged lymphopenia may contribute to viral and opportunistic infections. Rare patients may develop profound and persistent marrow dysfunction, including severe aplasia, requiring prolonged supportive care. The mechanisms are multifactorial and include previous cytotoxic exposure, marrow disease, lymphodepleting chemotherapy, inflammatory cytokine signaling, and impaired hematopoietic reserve [34].

#### 4.1.3. B-Cell Aplasia and Hypogammaglobulinemia

B-cell aplasia and plasma-cell depletion should be distinguished because their effects on humoral immunity depend partly on the therapeutic target. CD19-directed CAR-T cells deplete CD19-expressing naïve and memory B cells, impairing the generation of new antibody responses and B-cell-dependent immune memory [36,37]. However, many long-lived plasma cells lack CD19 and may persist after CD19-directed CAR-T therapy; clinical data indicate that some pre-existing antigen-specific antibody responses can be preserved despite B-cell aplasia [38]. By contrast, BCMA is expressed on plasma cells, and BCMA-directed CAR-T therapy can directly deplete antibody-producing plasma-cell populations, contributing to broader and sometimes prolonged hypogammaglobulinemia [39,40]. BCMA-directed BsAbs may also sustain plasma-cell impairment during ongoing treatment. Accordingly, loss of all pre-existing antibody titres should not be assumed after CD19-directed therapy, and B-cell aplasia should not be equated with direct plasma-cell depletion. Reduced antibody availability may compromise humoral protection against extracellular bacteria and viral pathogens [41].

#### 4.1.4. T-Cell Dysfunction

T-cell exhaustion is a progressive state of functional impairment that develops following prolonged antigen exposure or chronic T-cell stimulation. It can involve upregulation of inhibitory receptors such as PD-1, LAG-3, and TIM-3, reduced cytokine production, and impaired cytotoxicity [42,43,44]. In the context of CAR-T therapy and BsAbs, this literature often describes the function of therapeutic or repeatedly stimulated T cells. It should not automatically be interpreted as evidence of generalized impairment of the patient’s endogenous antiviral T-cell immunity. The degree to which treatment-associated T-cell exhaustion independently causes clinical infection susceptibility remains insufficiently defined. Infection risk should therefore be discussed alongside better-established contributors, including lymphopenia, corticosteroid exposure, cytopenias, B-cell or plasma-cell depletion, and cumulative immunosuppression [45].

### 4.2. Immunosuppressive Effects of Toxicity Management

A separate mechanism of infection susceptibility is represented by immunosuppressive treatment administered to control immune-mediated toxicities.

#### 4.2.1. Corticosteroids

Corticosteroids impair both innate and adaptive immunity by inhibiting T-cell activation and proliferation, disrupting antigen-presenting cell function, and suppressing inflammatory cytokine production [20,21]. The resulting infection risk depends on dose, treatment duration, cumulative exposure, and concomitant immunosuppression. Prolonged high-dose therapy is associated with an increased risk of opportunistic infections, particularly Pneumocystis jirovecii pneumonia (PJP), invasive fungal infections, and herpesvirus reactivation [20,21,22]. This is particularly relevant in CAR-T recipients receiving high-dose corticosteroids for immune effector cell-associated neurotoxicity syndrome (ICANS). Similarly, in patients treated with immune checkpoint inhibitors (ICIs), immunosuppressive therapy for immune-related adverse events (irAEs), rather than checkpoint blockade itself, may represent the major driver of opportunistic infections [20,21,22].

#### 4.2.2. Tocilizumab and Other Immunomodulators

Tocilizumab, an IL-6 receptor antagonist, is primarily used for CRS associated with CAR-T therapy. IL-6 blockade may attenuate inflammatory responses and potentially modify clinical manifestations of infection. However, its independent contribution to infectious risk is difficult to isolate because it is generally administered in patients who are already experiencing severe systemic inflammation and may also receive corticosteroids [46]. Other agents, including IL-1 inhibitors and JAK inhibitors, may further modify host immune responses when used for refractory CRS, ICANS, or other immune-mediated toxicities [47,48,49]. The infectious consequences of these agents should therefore be interpreted in the context of cumulative immunosuppression rather than attributed to a single drug.

#### 4.2.3. Patient- and Disease-Related Risk Modifiers

Infection risk in patients receiving cancer immunotherapy is influenced not only by treatment-related immune dysfunction but also by patient- and disease-specific factors. Advanced age, comorbidities, organ impairment, high tumor burden, bone marrow infiltration, and prior cytotoxic therapies can substantially compromise immune competence and hematopoietic reserve. In addition, previous radiotherapy, targeted agents, poor nutritional status, and a history of prior infections may further impair innate and adaptive immune responses [50,51].

## 5. Microbiological Spectrum

### 5.1. Bacterial Infections

Bacterial infections are among the most frequent complications following CAR-T cell and BsAbs, as well as in patients receiving ICIs [23]. Gram-negative pathogens such as *Escherichia coli* and *Klebsiella* spp., and Gram-positive bacteria including *Clostridioides difficile*, *Staphylococcus* spp., and *Streptococcus mitis*, are commonly reported [24,25]. Early post-therapy infections typically occur within the first 30 days, when innate immune defenses are most compromised, while late infections are often associated with prolonged B-cell aplasia and hypogammaglobulinemia [26]. In patients receiving ICIs for solid tumors, bacterial infections are generally less frequent than with cytotoxic therapies or intensive cellular therapies but remain an important clinical concern, particularly in real-world practice. In retrospective cohorts of solid tumor patients treated with PD-1/PD-L1 or CTLA-4 inhibitors, overall infection rates vary widely (≈19–27%), with bacterial pathogens accounting for a substantial proportion of documented episodes [52]. In one multicenter analysis of 111 patients with melanoma, lung, and genitourinary cancers treated with ICIs, suspected or confirmed bacterial infections occurred in approximately 24% of patients, with respiratory tract and genitourinary sites among the most common foci [21].

### 5.2. Antimicrobial Resistance and MDR Infections

Direct clinical evidence on antimicrobial resistance (AMR) in patients receiving cancer immunotherapy remains limited and is derived mainly from CAR-T recipients and broader hematology and cellular-therapy cohorts. In a retrospective, single-center study of CAR-T recipients, 35 patients who developed carbapenem-resistant organism (CRO) infections within one year were compared with 280 controls without CRO infection. Previous CRO colonization was strongly associated with subsequent CRO infection (odds ratio [OR] 15.48, 95% CI 6.43–37.25), while CRO infection was associated with worse one-year outcomes (hazard ratio [HR] 4.40, 95% CI 2.32–8.37) [4]. Although these findings support the importance of reviewing previous colonization and microbiological results before initiating empirical antimicrobial therapy, their interpretation is limited by the observational design and potential differences in local epidemiology and patient characteristics.

Similarly, a 2026 cohort of 182 patients undergoing cellular therapies found an association between previous colonization and subsequent colonization with carbapenem-resistant Gram-negative bacilli and vancomycin-resistant enterococci (VRE). Among colonized patients, microbiological concordance with subsequent bloodstream infection was observed in 3 of 16 patients colonized with carbapenem-resistant Gram-negative bacilli and 2 of 45 colonized with VRE [5]. However, 85% of this cohort underwent allogeneic hematopoietic cell transplantation (HCT), limiting the extent to which these findings can be extrapolated specifically to CAR-T recipients. For empirical antimicrobial selection, the most relevant factors remain previous isolation or colonization with resistant organisms, recent and cumulative antibiotic exposure, clinical severity, prior healthcare exposure, and local antimicrobial susceptibility patterns (antibiograms). Potential pathogens of concern include extended-spectrum β-lactamase (ESBL)-producing *Enterobacterales,* carbapenem-resistant Enterobacterales and other carbapenem-resistant Gram-negative bacilli, VRE, and methicillin-resistant *Staphylococcus aureus* (MRSA), depending on local epidemiology. Importantly, colonization increases the likelihood of subsequent infection with resistant organisms in some settings, but does not constitute active infection and should not, in itself, prompt empirical treatment with the broadest-spectrum antibiotics. Appropriate cultures should be obtained promptly when indicated, empirical therapy should be regularly reassessed as microbiological and clinical data become available, and treatment should be narrowed whenever clinically appropriate [50]. Evidence on AMR incidence and clinical outcomes in patients receiving BsAbs or ICIs remains particularly scarce. Consequently, infection rates reported in these populations cannot be used to infer treatment-specific AMR rates.

### 5.3. Viral Infections: CMV, EBV, HSV, VZV, HHV-6, HBV

Herpesviruses, including cytomegalovirus (CMV), Epstein–Barr virus (EBV), herpes simplex virus (HSV), human herpesvirus 6 (HHV-6), and varicella-zoster virus (VZV), are relevant after CAR-T and BsAb therapy, although reported incidence varies across targets, prior therapies, prophylaxis, and follow-up [29,30,31,32,53]. CMV reactivation may be of particular concern in selected patients receiving BCMA-directed therapies or substantial corticosteroid exposure. In patients receiving ICIs, respiratory viruses remain common, whereas CMV and VZV reactivation are less typical during uncomplicated monotherapy and become more relevant with secondary immunosuppression. Hepatitis B virus (HBV) screening and appropriate prophylaxis or pre-emptive monitoring remain important before ICI initiation in patients with evidence of current or previous HBV infection [29,30,31,32,53]. Practical CMV and HHV-6 surveillance considerations are addressed separately in Section 6.3.

### 5.4. Fungal and Opportunistic Infections

Invasive fungal infections (IFIs) are less frequent than bacterial infections but are associated with substantial morbidity and mortality. After CAR-T therapy, reported IFI rates vary widely because of differences in prophylaxis, definitions, follow-up, and patient selection; Aspergillus and Candida are among the most frequently reported pathogens [54,55]. Risk is greatest with prolonged severe neutropenia, high-dose corticosteroids, multiple immunosuppressive agents, and persistent lymphopenia.

PJP deserves separate consideration. Although uncommon in some CAR-T cohorts, PJP has been reported after both CAR-T and BsAb therapy, particularly when prophylaxis is interrupted or CD4 recovery is delayed. Expert consensus for patients with MM receiving BsAbs recommends PJP prophylaxis during treatment [27]. For CAR-T recipients, trimethoprim-sulfamethoxazole is generally initiated after the early post-infusion period and continued until adequate immune recovery, with a CD4 count above 200 cells/μL commonly used as one practical milestone; local protocols and individual risk may require a different duration [28]. In patients receiving ICIs, PJP and invasive fungal infections should primarily be considered complications of secondary immunosuppression rather than direct effects of checkpoint blockade. They warrant consideration in patients receiving prolonged high-dose corticosteroids, anti-TNF therapy, or other immunosuppressive regimens, especially when respiratory symptoms are atypical or fail to respond to conventional antibacterial treatment [56].

## 6. Diagnosis and Multidimensional Risk Stratification

The diagnosis of infection after immunotherapy is complicated by overlapping manifestations of infection, CRS, ICANS, and irAEs. Fever, hypotension, inflammatory marker elevation, pulmonary infiltrates, and neurological symptoms may have multiple competing explanations. A low threshold for microbiological investigation is therefore appropriate in high-risk patients, particularly when clinical evolution is atypical or fails to follow the expected course of treatment-related toxicity.

### 6.1. Hematologic Recovery and Immune Milestones

Definitions of hematologic recovery should be explicit. After CAR-T therapy, recovery should not be equated with a single normal blood count; clinically meaningful assessment includes serial absolute neutrophil count (ANC), platelet, and hemoglobin trajectories and the absence of prolonged immune effector cell-associated hematotoxicity (ICAHT). Early ICAHT is assessed during days 0–30, whereas late ICAHT is assessed after day +30 [57]. For consistency, severe neutropenia is defined here as ANC < 500 cells/μL and profound neutropenia as ANC < 100 cells/μL; duration of neutropenia is also clinically important, and these terms should not be used interchangeably. For PJP prevention, CD4+ T-cell recovery is often used as an operational milestone, with >200 cells/μL commonly adopted when considering discontinuation of prophylaxis. This threshold should not be interpreted in isolation: persistent corticosteroid exposure, ongoing BsAb therapy, severe lymphopenia, or other immunosuppressive conditions may justify continued prophylaxis despite a CD4 count above 200 cells/μL [27,28].

### 6.2. Risk Scores and Biomarkers

No universally validated infection-specific risk score currently exists across CAR-T, BsAb, and ICI therapies. For CAR-T recipients, the CAR-HEMATOTOX score provides a clinically useful estimate of hematotoxicity risk and may indirectly identify patients at increased infectious risk because prolonged neutropenia is a major determinant of infection [57]. However, it is not an infection score and should not be presented as such. Candidate biomarkers include baseline and serial IgG, lymphocyte subsets, CD4/CD8 counts, neutrophil trajectories, inflammatory markers, ferritin, CRP, and markers of immune reconstitution. Their clinical value is currently strongest when used longitudinally rather than as isolated baseline measurements. For ICIs, no validated prospective infection-prediction model has been established. Future models should integrate treatment intensity, corticosteroid exposure, prior colonization, microbiological history, organ dysfunction, and dynamic immune parameters.

### 6.3. CMV and HHV-6 Monitoring

CMV surveillance after CAR-T remains heterogeneous across centers. Baseline serostatus and risk-adapted quantitative PCR monitoring may be considered, particularly in CMV-seropositive patients receiving BCMA-directed therapy or substantial corticosteroid exposure [53]. Universal prolonged monitoring for every recipient is not supported by robust prospective evidence, and the optimal threshold for pre-emptive treatment remains uncertain; results should be interpreted according to viral kinetics, symptoms, immune status, and local assay characteristics. HHV-6 testing should be clinically driven, particularly for unexplained encephalopathy, seizures, or atypical neurological deterioration in patients with prolonged immunosuppression. These decisions should be integrated with the overall clinical picture rather than based on isolated low-level viral detection.

## 7. Prevention and Management

### 7.1. Antimicrobial Prophylaxis

Prophylaxis should be tailored to the expected immune defect, its duration, and the patient’s additional risk factors. HSV/VZV prophylaxis with acyclovir or valacyclovir is standard for appropriate CAR-T recipients, while PJP prophylaxis is generally initiated after the early post-infusion period and continued until adequate immune recovery, according to local protocols [28]. In patients receiving MM BsAbs, antiviral and PJP prophylaxis are key components of expert-consensus supportive care, together with immunoglobulin monitoring [27]. Antibacterial prophylaxis after CAR-T therapy should be individualized. It may be considered in patients at risk of prolonged severe neutropenia, those with a history of previous infections, or those with other high-risk features, taking into account local resistance patterns and institutional protocols. However, universal fluoroquinolone prophylaxis for all CAR-T recipients is not currently established. In a 2026 retrospective cohort of 204 patients managed without routine fluoroquinolone prophylaxis, bloodstream infections occurred in 3.4% of patients. Factors associated with early infection included axicabtagene ciloleucel treatment, grade ≥ 3 ICANS, and prolonged neutropenia [58]. Although this single-cohort finding does not establish a universal policy, it highlights the need to balance potential benefits against the risks of antimicrobial resistance, Clostridioides difficile infection, and microbiome disruption. Mold-active prophylaxis may be considered when severe neutropenia (ANC < 500 cells/μL) is expected to persist for more than seven days, particularly in patients receiving prolonged or high-dose corticosteroids or with a history of invasive fungal infection, persistent lymphopenia, or other major risk factors [12,28,57]. Empirical treatment of febrile episodes should take into account previous resistant isolates, recent antimicrobial exposure, local resistance patterns, clinical severity, and available microbiological results, with prompt reassessment and de-escalation when appropriate [4,50].

### 7.2. Immunoglobulin Replacement Therapy

The evidence for immunoglobulin replacement therapy (IGRT) is strongest in patients with prolonged B-cell or plasma-cell depletion and hypogammaglobulinemia, particularly after CAR-T and BCMA-directed BsAbs. Many consensus recommendations use IgG < 400 mg/dL and/or recurrent or severe bacterial infections as practical indications [27,28,59]. Intravenous immunoglobulin (IVIG) at approximately 400 mg/kg every 3–4 weeks, or individualized subcutaneous immunoglobulin replacement, can be adjusted according to trough IgG concentrations and clinical response.

Importantly, IGRT should not be described as universally indicated for all patients with a low IgG value. The decision should consider infection history, depth and duration of hypogammaglobulinemia, ongoing therapy, vaccination responses, and competing risks. Recent evidence from the evolving BsAb/CAR-T literature supports early, individualized replacement in high-risk patients, but prospective randomized evidence remains limited [60].

### 7.3. Vaccination Strategies and Timing

Vaccination should ideally be optimized before treatment whenever clinically feasible, because responses are generally more robust before lymphodepletion or profound immune dysfunction. Before CAR-T therapy, seasonal inactivated influenza and SARS-CoV-2 vaccines are the principal vaccines that may be considered shortly before treatment when timing permits; other vaccines should be planned according to immunization history and the expected treatment date [28,33,61,62,63]. After CAR-T therapy, vaccine timing should be vaccine-specific rather than based on a universal three-month start. The American Society for Transplantation and Cellular Therapy (ASTCT) guidance allows influenza and SARS-CoV-2 vaccination to be considered from approximately three months after CAR-T because of their seasonal and public-health importance. Broader non-live revaccination, including pneumococcal, tetanus/diphtheria/pertussis, and hepatitis A/B vaccines when indicated, is generally considered from at least six months after CAR-T in appropriate patients, taking account of immune recovery, subsequent therapy, and clinical status [28]. Recombinant zoster vaccine timing should follow the applicable guidance and individual immune status; live attenuated vaccines should be avoided during substantial immunosuppression and considered only after specialist assessment confirms adequate immune reconstitution. For patients receiving BsAbs, vaccination before treatment is preferable when feasible because ongoing B-cell or plasma-cell targeting may reduce humoral responses. Non-live vaccines may still be administered during ongoing therapy when indicated, but expected immunogenicity should be discussed and vaccination should not replace other preventive measures [27,63]. In patients receiving ICIs, non-live vaccination is generally feasible and should not be unnecessarily delayed; influenza, pneumococcal, SARS-CoV-2, recombinant zoster, and other age- or risk-based vaccines should be considered according to national guidance and concomitant immunosuppressive treatment [33,61,62,63]. See the Table 2 for the main pactical prevention milestones.

**Table 2:** Practical prevention milestones.

ICIs	BsAbs	CAR-T	Milestone
Age/risk-based vaccines; HBV/TB assessment per epidemiology and planned immunosuppression [33,61]	Vaccinate before treatment if feasible; HBV screen; baseline IgG [27,63]	Screen relevant infections; document CMV/HBV status; update vaccines when feasible [28,33]	Before therapy
No routine prophylaxis unless immunosuppression or another risk factor is present [22,29]	PJP/antiviral prophylaxis; IgG monitoring and early infection vigilance [27]	HSV/VZV and PJP prophylaxis; consider antibacterial/mold prophylaxis for selected high-risk patients [28,58]	Early treatment
Reassess risk based on steroid dose/duration and other immunosuppressants [22,29]	Continue prophylaxis during ongoing exposure; repeat IgG and replace when indicated [27]	PJP prophylaxis until CD4 recovery (commonly >200/μL); continue antiviral prophylaxis per protocol; assess IgG [27,28]	Immune recovery
Non-live vaccines generally feasible; follow age/risk recommendations without unnecessary delay [33,61,62,63]	Individualized; vaccinate before treatment if feasible; non-live vaccines may be offered during treatment, although responses may be reduced [27,63]	Influenza/SARS-CoV-2 from ~3 months; broader non-live revaccination generally ≥6 months when appropriate; live vaccines only after specialist review [28,33]	Vaccination after therapy

Abbreviations: BsAbs: Bispecific antibodies; CAR-T: Chimeric Antigen Receptor T-cell therapy; CD4: Cluster of Differentiation 4; CMV: Cytomegalovirus; HBV: Hepatitis B Virus; HSV: Herpes Simplex Virus; ICIs: Immune Checkpoint Inhibitors; IgG: Immunoglobulin G; PJP—*Pneumocystis jirovecii* pneumonia; TB: Tuberculosis; VZV: Varicella-Zoster Virus.

## 8. Future Directions

Future research should prioritize prospective multicenter cohorts with standardized definitions of infection, severe infection, infection-related hospitalization, infection-attributable mortality, MDR colonization, and MDR infection. At minimum, studies should record the underlying malignancy, therapeutic target and product, previous treatment lines, neutropenia and lymphopenia trajectories, immunoglobulin concentrations, corticosteroid exposure, prophylaxis, previous resistant colonization or infection, antimicrobial class and duration, microbiological susceptibility, and follow-up. Results should be reported separately by treatment platform and disease indication wherever sample size permits. The current AMR evidence base is dominated by CAR-T and broader hematology/cellular-therapy cohorts; robust incidence and outcome data for BsAb- and ICI-treated patients remain a priority. Studies should also distinguish colonization from clinical infection and evaluate whether resistance-directed management improves patient outcomes without producing unnecessary broad-spectrum antimicrobial exposure.

At the clinical-program level, a practical infection and AMR pathway could incorporate a baseline review of prior microbiology and antibiotic exposure, local antibiogram-informed empirical-treatment algorithms, clear criteria for starting and stopping prophylaxis, vaccination milestones, and predefined triggers for infectious-disease and antimicrobial-stewardship consultation. Prospective evaluation should include patient-centered outcomes and antimicrobial-use measures, including broad-spectrum antibiotic days, de-escalation, *Clostridioides difficile* infection, and newly detected MDROs. This implementation agenda is distinct from treatment-platform risk prediction: it tests whether structured pathways improve care in real-world settings while preserving timely treatment of suspected infection.

## 9. Conclusions

Infectious complications of CAR-T cells, BsAbs, and ICIs reflect distinct patterns of immune dysfunction, disease context, treatment exposure, and supportive care. CAR-T and BCMA-directed BsAbs may be associated with sustained infectious risk due to cytopenias, B-cell or plasma-cell depletion, and hypogammaglobulinemia. In contrast, infections during ICI treatment are often shaped by concomitant chemotherapy or immunosuppression for irAEs. Assess AMR risk using prior microbiology, antimicrobial exposure, healthcare contact, and local resistance patterns rather than inferring it from the immunotherapy platform alone. Current AMR data are strongest for CAR-T and broader cellular-therapy cohorts, and evidence remains limited for BsAbs and ICIs. Targeted prophylaxis, individualized vaccination, appropriate immunoglobulin replacement, and antimicrobial stewardship should be integrated into prevention pathways while acknowledging uncertainties and avoiding unnecessary antimicrobial exposure.

## Data Availability

No new data were created or analyzed in this study. Data sharing is not applicable to this article.
